# Deep learning-based algorithm accurately classifies sleep stages in preadolescent children with sleep-disordered breathing symptoms and age-matched controls

**DOI:** 10.3389/fneur.2023.1162998

**Published:** 2023-04-14

**Authors:** Pranavan Somaskandhan, Timo Leppänen, Philip I. Terrill, Sigridur Sigurdardottir, Erna Sif Arnardottir, Kristín A. Ólafsdóttir, Marta Serwatko, Sigurveig Þ. Sigurðardóttir, Michael Clausen, Juha Töyräs, Henri Korkalainen

**Affiliations:** ^1^School of Information Technology and Electrical Engineering, The University of Queensland, Brisbane, QLD, Australia; ^2^Department of Technical Physics, University of Eastern Finland, Kuopio, Finland; ^3^Diagnostic Imaging Center, Kuopio University Hospital, Kuopio, Finland; ^4^Reykjavik University Sleep Institute, School of Technology, Reykjavik University, Reykjavik, Iceland; ^5^Internal Medicine Services, Landspitali–The National University Hospital of Iceland, Reykjavik, Iceland; ^6^Department of Clinical Engineering, Landspitali University Hospital, Reykjavik, Iceland; ^7^Department of Immunology, Landspitali University Hospital, Reykjavik, Iceland; ^8^Faculty of Medicine, University of Iceland, Reykjavik, Iceland; ^9^Department of Allergy, Landspitali University Hospital, Reykjavik, Iceland; ^10^Children's Hospital Reykjavik, Reykjavik, Iceland; ^11^Science Service Center, Kuopio University Hospital, Kuopio, Finland

**Keywords:** Pediatric sleep staging, preadolescent cohort, inter-rater reliability, pediatric sleep-disordered breathing, community controls, deep learning, recurrent neural network, convolutional neural network

## Abstract

**Introduction:**

Visual sleep scoring has several shortcomings, including inter-scorer inconsistency, which may adversely affect diagnostic decision-making. Although automatic sleep staging in adults has been extensively studied, it is uncertain whether such sophisticated algorithms generalize well to different pediatric age groups due to distinctive EEG characteristics. The preadolescent age group (10–13-year-olds) is relatively understudied, and thus, we aimed to develop an automatic deep learning-based sleep stage classifier specifically targeting this cohort.

**Methods:**

A dataset (*n* = 115) containing polysomnographic recordings of Icelandic preadolescent children with sleep-disordered breathing (SDB) symptoms, and age and sex-matched controls was utilized. We developed a combined convolutional and long short-term memory neural network architecture relying on electroencephalography (F4-M1), electrooculography (E1-M2), and chin electromyography signals. Performance relative to human scoring was further evaluated by analyzing intra- and inter-rater agreements in a subset (*n* = 10) of data with repeat scoring from two manual scorers.

**Results:**

The deep learning-based model achieved an overall cross-validated accuracy of 84.1% (Cohen’s kappa κ = 0.78). There was no meaningful performance difference between SDB-symptomatic (*n* = 53) and control subgroups (*n* = 52) [83.9% (κ = 0.78) vs. 84.2% (κ = 0.78)]. The inter-rater reliability between manual scorers was 84.6% (κ = 0.78), and the automatic method reached similar agreements with scorers, 83.4% (κ = 0.76) and 82.7% (κ = 0.75).

**Conclusion:**

The developed algorithm achieved high classification accuracy and substantial agreements with two manual scorers; the performance metrics compared favorably with typical inter-rater reliability between manual scorers and performance reported in previous studies. These suggest that our algorithm may facilitate less labor-intensive and reliable automatic sleep scoring in preadolescent children.

## Introduction

Sleep is a vital component of health and well-being for children and is particularly important for maintaining normal neurocognitive functions (1–4). Subsequently, sleep disorders are associated with detrimental health consequences such as emotional and behavioral problems (5, 6) and attention deficiency (7). Given that sleep disorders such as obstructive sleep apnea (OSA) are common in children (prevalence of 1%–4%) (8), there is substantial motivation to develop efficient and effective diagnostic systems. Accurate sleep stage classification is an important step in both the diagnosis of pediatric sleep disorders and research investigating normal physiological sleep; and is manually scored according to the American Academy of Sleep Medicine (AASM) (9) guidelines using electroencephalography (EEG), electrooculography (EOG), and submental electromyography (EMG) signals recorded using polysomnography (PSG) (9). However, manual sleep scoring is expensive and time-consuming (10) and is subjective leading to inconsistency between human scorers (11–17). While the typical Cohen’s kappa for inter-rater agreement is 0.76–0.78 in adults, it can be as low as 0.57–0.63 between international sleep centers (11, 12); and could be even lower in children due to greater variability in EEG signal characteristics (18–21).

Automated sleep staging systems have been proposed to overcome the limitations of manual sleep stage classification; and such algorithms are already incorporated in some commercial PSG software where they provide a preliminary scoring that is verified and corrected by a human expert. Numerous published studies have also attempted to fully automate the sleep staging process (22–47). Whilst historically, these have used feature engineering approaches or hand-crafted rules (29–32), most recent studies utilize deep learning-based algorithms (22–26, 33–46). Although modern deep learning-based approaches generally perform well (kappa agreement typically ranging between 0.67 and 0.87) (48–51), the majority have focused on adult populations (22, 23, 30–39, 41, 43, 45). Due to the continuous maturation of the brain, EEG signals in children may vary with age (18–20); and therefore, it is uncertain whether the sophisticated sleep staging systems designed for adults generalize well to children.

A smaller number of recent studies have focused on automatic sleep staging in children (24–29, 40, 42, 44, 47). Whilst some of these focus on two- or three-stage sleep classification (24, 26–28) [predominantly those considered infants (26–28)] or using non-EEG-based approaches intended for limited channel screening (29, 47), studies published in parallel with the development of this work using electrophysiological channels have demonstrated high sleep classification performance (40, 42, 44). However, there are some important limitations. Firstly, none of these studies included both children with sleep disorders and asymptomatic controls recruited from the community. Secondly, there are substantial gaps in the ages of the children studied. In particular, the preadolescent children (10–13-year-olds) are not well represented, reflecting them being a relatively understudied group in sleep research more generally. Given the substantial emotional and hormonal changes (52) during this period having an automated tool to better facilitate the investigation of physiological and pathophysiological sleep in this age group is highly desirable.

As such, the overarching aim of this study was to develop a deep learning-based method to automate sleep stage classification, specifically targeting preadolescent children with sleep-disordered breathing (SDB) symptoms and age and sex-matched community controls. We hypothesized that a combined convolutional and long short-term memory network architecture enables accurate pediatric sleep stage classification using raw frontal EEG, EOG, and EMG signals. This algorithm was developed and cross-validated using a dataset of overnight PSG recordings of Icelandic children. Performance relative to human scoring was further evaluated by conducting intra-rater and inter-rater agreement analysis in a subset of data with repeated scorings from two experienced human scorers.

## Methods

### Dataset

The dataset utilized in this study comprised 10–13 years old Icelandic children from the EuroPrevall-iFAAM birth cohort (53–56). Of the Icelandic EuroPrevall (57) study population, children who were reported to snore at least three times or have witnessed apneas at least once a week (*n* = 109) were invited to engage in a home PSG. Out of the 109 invitees, 55% agreed to participate (*n* = 60). Additionally, 58 children with no snoring or apneas were included in the age and sex-matched control group. Two of the recordings were not completed successfully, and one participant declined the full usage of data. Thus, the total study population included 115 children with almost equal proportions of SDB-symptomatic (*n* = 59) and control participants (*n* = 56).

Informed written consent was obtained from parents or legal guardians for all children who participated in this study; and data collection was approved by the Ethical Committee of Landspitali—the National University Hospital of Iceland and the National Bioethics Committee of Iceland (VSN 18–206). The PSG device used for this study was Nox A1 (Nox Medical, Reykjavik, Iceland) and was configured by two experienced sleep technologists. All the PSG recordings were conducted at home over a single night. The sleep stages of all 115 PSGs included in the final study population were initially scored once manually into categories: W, N1, N2, N3, and R by one of two human scorers using the full montage of recommended channels in compliance with current AASM guidelines (9). This scoring was treated as the “gold standard” and utilized as the reference to compare with during the neural network training, validation, and testing.

In addition, a subset of this data comprising 10 PSGs was rescored once more by the same manual scorer and twice separately by the other scorer. This yielded a total of four distinct scorings, used solely for the purpose of conducting a separate comparative intra- and inter-rater agreement analysis. This was conducted to demonstrate the reliability of our algorithm by investigating whether our results are comparable to inter- and intra-rater reliability between manual scorers.

### Software and hardware configurations for algorithm development and data-analysis

We used Conda (version 4.8.3) environment with Python 3.6.10, Keras API (version 2.3.1), and TensorFlow (version 2.2.0) backend to implement the neural network architecture. The training was conducted using an AMD Ryzen Threadripper 2990WX CPU, x86_64 architecture, 128 GB RAM, and NVIDIA GeForce RTX 2080 GPU. Statistical analyses related to intra-rater and inter-rater reliabilities were conducted in Python 3.6.10 with scikit-learn 0.24.2.

### Neural network architecture

We adopted an architecture comprised of a combined convolutional neural network (CNN) and recurrent neural network (RNN) trained in an end-to-end manner that we have previously utilized for automated sleep staging in adult populations (22). The CNN part was chosen to study the unique features of the sleep stages, while the RNN was utilized to learn the temporal distribution. This and similar architectures (i.e., variations of CNN-RNN combined networks) have previously demonstrated competitive results in adult sleep staging (22, 35, 37, 39); and part of our motivation was to examine how generalizable such an architecture is to children in the preadolescent age group.

**Figure 1:**
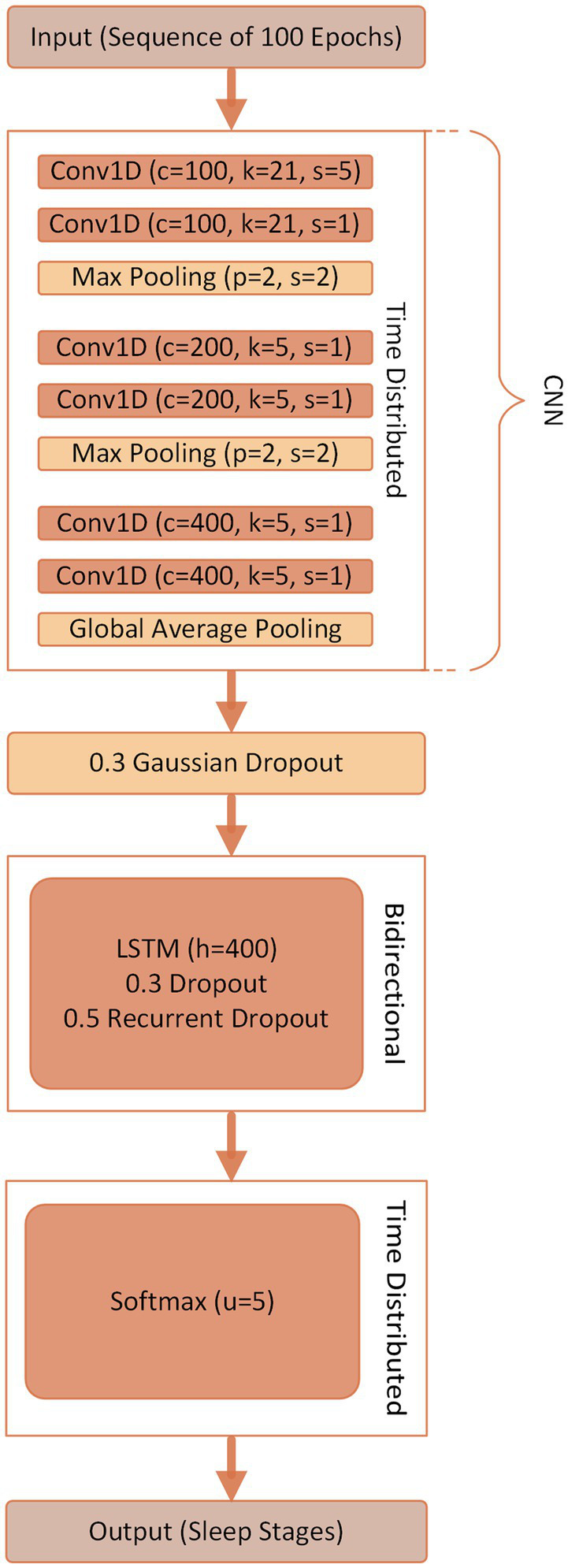
Illustration of the combined convolutional neural network (CNN) and long short-term memory (LSTM) network architecture. The parameters of the 1D convolution layers (Conv1D) are provided as (c = number of convolutional filters, k = kernel size, s = stride size). For the max pooling, the parameters are given as (p = pool size, s = stride size). LSTM and the softmax dense layer have the number of units as the parameter (i.e., h = number of hidden/output units in LSTM and u = number of nodes in dense layer). The dropout layers were active only during the training phase. A sequence of softmax values was generated by the model indicating the probabilities of possible sleep stages for every epoch. The sleep stage with the highest softmax value was estimated as the corresponding sleep stage of that epoch.

The CNN part comprised six 1D convolution layers, each of which was followed by batch normalization and a rectified linear unit (ReLU) activation function. Two max-pooling layers and a global average pooling layer were included in the architecture, each situated after every two 1D convolutional layers, respectively (Figure 1). The complete network consisted of a time-distributed layer of the entire CNN part, followed by a gaussian dropout layer, a bidirectional long short-term memory (LSTM) layer, and a time-distributed dense layer with softmax activation (Figure 1). A tanh activation function was used in the LSTM, and a hard-sigmoid activation was used in the recurrent step. The final layer of the complete architecture was a dense layer accompanied by a softmax activation function generating the output sequence of the sleep stage probabilities.

### Automatic sleep staging

Three channels consisting of frontal EEG (derivation F4-M1), EOG (derivation E1-M2), and submental EMG (derivation Chin1-Chin2) were used as the input for the final neural network architecture. The primary motivations for using these channels were: (1) use of frontal channels to make it more practical for the ambulatory sleep settings and simplify the overall measurement protocol; (2) to maintain consistency with recent literature that is tended towards using minimal channels to perform accurate sleep staging utilizing deep learning techniques; and (3) for consistency with the AASM criteria (9), which explicitly requires EEG, EOG, and EMG signals for sleep stage classification. These signals were initially recorded with a sampling frequency of 200 Hz but downsampled to 100 Hz to reduce the computational load. Signal segments at the beginning and the end of the recordings without manual scorings were excluded from the final analysis.

The complete dataset was initially divided into two individual sets: (1) Analysis set: primary data, which comprised 105 PSGs scored once manually and used for the neural network training, validation, and testing; and (2) Comparison set: which included the remaining 10 PSGs that were manually scored four times, i.e., twice each by two human experts. The comparison set was held out of training and evaluation of the model; and solely used for investigating intra- and inter-rater agreements between the two independent manual scorers and relative to the automated classification.

**Figure 2:**
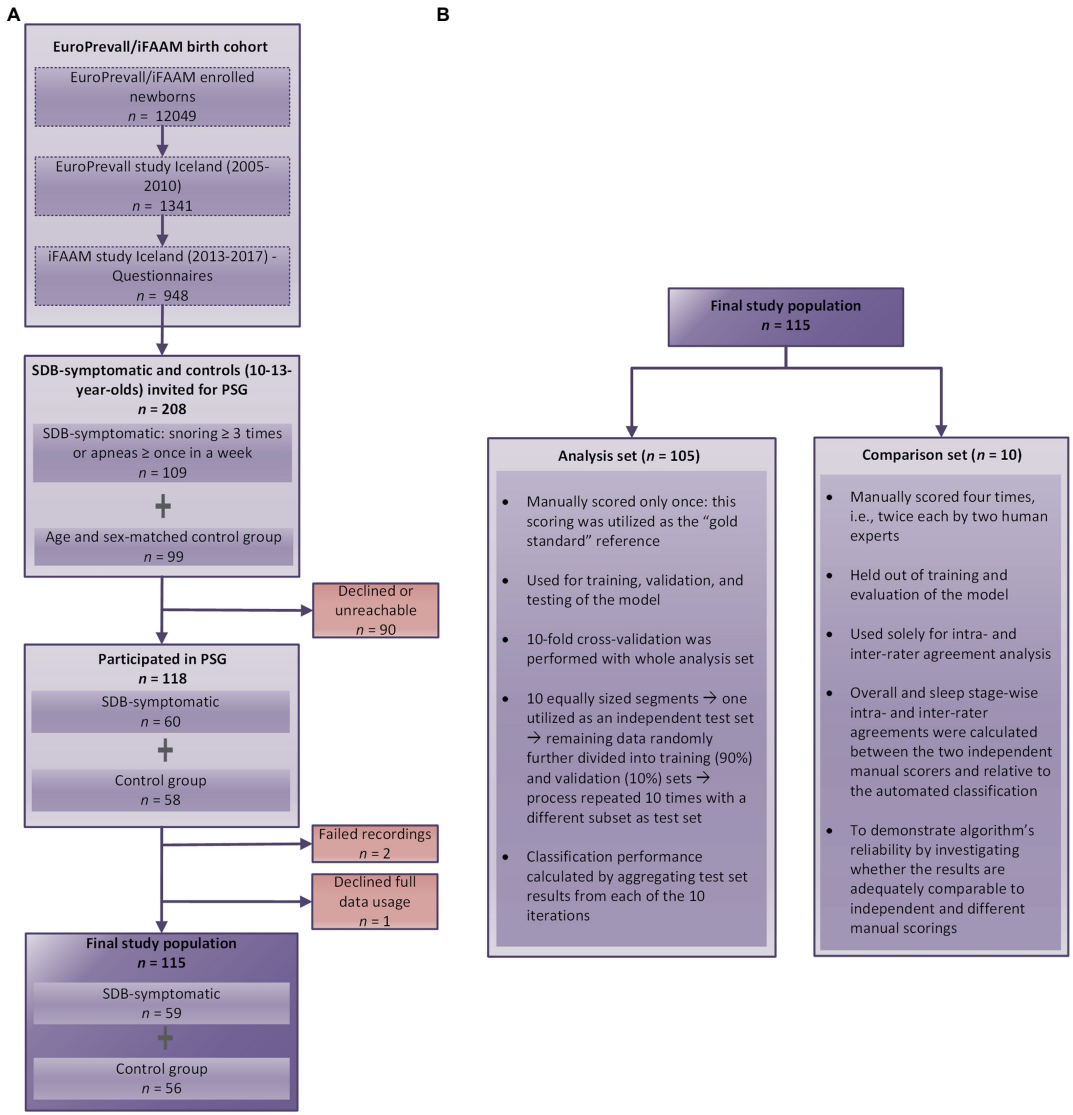
Data flow diagram that illustrates **(A)** how the final study data was formed and **(B)** how the data was divided and used for the analyses. PSG, polysomnography; EEG, electroencephalography.

10-fold cross-validation was performed with the whole analysis set (*n* = 105) to obtain the best estimate of the non-biased model performance. For the cross-validation, the analysis set was first randomly separated into 10 equally sized segments. One of these segments was utilized as an independent test set, while the remaining data were further randomly divided into training (90%) and validation (10%) sets to train and choose the optimal model. The test set was held intact from the model training and validation and used as an unseen data for the model evaluation. This entire process was repeated 10 times with a different subset representing the independent test set in each iteration. The final reported results are for the classification performance in the aggregation of the test set from each of the 10 iterations of the cross-validation (*n* = 105). Figure 2 presents a data flow diagram, which illustrates how the final study data was formed and how it was divided and used for the analysis.

The model was trained in a sequence-to-sequence manner with an input sequence length of one hundred 30-s epochs, i.e., an input sequence of one hundred epochs was mapped to the target reference sleep stage sequence of identical length at once to comprehend inter-epoch dependency. The sequence length was chosen based on initial testing and as a compromise between computational load and capturing a sufficiently long sleep cycle. A categorical cross-entropy loss function, an Adam optimizer with warm restarts (58), and a learning rate range of 0.001 to 0.00001 optimized with a learning rate finder (59) were used during training. In the training set, an overlap of 75% was used to multiply the size of the training data by four when forming the sequences. No overlap was applied to validation or test sets. The maximum number of training epochs was set to 200. However, the training was only conducted until the validation loss function value no longer decreased considerably. For this, an early stopping callback with a patience coefficient of 20 was used, meaning that if validation loss did not improve for 20 consecutive epochs, then the training was stopped. This was done to prevent overfitting and to avoid wasting computational resources on training a model that is unlikely to improve. The final performance of the classifier was obtained by aggregating the test set results across all 10 folds. The accuracies were evaluated in an epoch-by-epoch manner. As an output of the model, the estimated sleep stage was determined to be the one with the highest softmax value. Cohen’s kappa coefficient (κ) (60) was utilized to assess the scoring consensus between manual and automatic scorings. Finally, we investigated the model performance separately between the SDB-symptomatic and control groups as well as between PSG-quantified clinical pediatric OSA (AHI ≥ 1) and non-OSA (AHI < 1). Groupwise performance was assessed by aggregating the test set results across all 10 folds and separately calculating the accuracies and kappa coefficients for each group.

For comparison with previous literature, we also separately trained and cross-validated our model to classify sleep into four (W/N1 + N2/N3/R) and three (W/N1 + N2 + N3/R) stages utilizing the analysis set as a secondary analysis. To determine inter-rater agreement-related performance between the automatic classifier and two manual annotators in the comparison set, we retrained the network using the entire analysis set and evaluated it on the unseen comparison set.

### Intra- and inter-rater agreement analysis

As a secondary investigation, we performed a separate intra- and inter-rater agreement analysis to examine the reliability of the neural network model by evaluating its predictive performance relative to multiple human scorings. A subset (i.e., the comparison set, *n* = 10, not included in the cross-validated training and evaluation) of the pediatric dataset was utilized for this analysis. Two European Sleep Research Society-certified sleep technologists from Reykjavik University Sleep Institute each scored the 10 PSGs twice (separated by at least 2 weeks); thus, producing four different sets of sleep scoring. Scorers were blinded to patient identities throughout the analysis. The manual scoring was compared with each other and with the neural network-predicted scores to evaluate the intra- and inter-rater reliabilities. In addition, we also examined how the automatic sleep stage classifications compared with the manual scoring when considering only the epochs that achieved a scoring consensus between both human scorers.

Score match percentage (percent accuracy) and kappa coefficient were used to determine the overall intra- and inter-rater agreements between different scorings. Sleep stage-specific intra- and inter-rater agreements were also calculated. Stage-specific agreements between the manual and automatic classifications were calculated with the manual scoring defined as the reference. Stage-specific agreements between manual classifications were defined as the average of the agreements calculated when each of the manual classifications was separately treated as the reference.

## Results

### Characteristics of the study population

A summary of demographic information and characteristics of the whole study population (*n* = 115), SDB-symptomatic subgroup (*n* = 59), and asymptomatic subgroup (*n* = 56) is presented in Table 1.

**Table 1:** The demographics and characteristics of the study population.

	Whole population	SDB-symptomatic group	Control group
*n* (boys %)	115 (66.1%)	59 (67.8%)	56 (64.3%)
Age (years), mean ± SD	11.8 ± 0.8	11.7 ± 0.8	11.9 ± 0.8
BMI (kg/m^2^), median (range)	19.7 (13.5–31.9)	20.6 (15.5–28.7)	18.9 (13.5–31.9)
AHI (events/h), median (range)	0.5 (0.0–6.3)	0.6 (0.0–6.3)	0.3 (0.0–3.2)
TST (min), mean ± SD	479.6 ± 54.1	471.3 ± 59.7	488.3 ± 46.5
Sleep efficiency (%), mean ± SD	93.1 ± 4.2	93.1 ± 4.0	93.1 ± 4.4

SD, Standard deviation; BMI, Body mass index; AHI, Apnea-hypopnea index; TST, Total sleep time; SDB, Sleep-disordered breathing.

Table 2 depicts the number and the percentage of 30-s epochs of each sleep stage in the whole dataset according to the manual reference scoring.

**Table 2:** Number and percentage of 30-s epochs of each sleep stage in the pediatric dataset based on initial manual reference scoring.

Sleep stage	Whole dataset (*n* = 115)		SDB-symptomatic group (*n* = 59)		Control group (*n* = 56)	
	Number	Percentage (%)	Number	Percentage (%)	Number	Percentage (%)
W	8,469	7.1	4,455	7.4	4,014	6.8
N1	3,724	3.1	1,657	2.8	2,067	3.5
N2	28,880	24.3	14,170	23.6	14,710	25.0
N3	54,786	46.1	28,055	46.8	26,731	45.5
R	22,917	19.3	11,649	19.4	11,268	19.2
Total	118,776	100	59,986	100	58,790	100

W, Wakefulness; R, Rapid eye movement sleep; N1, N2, N3, Three different levels of non-rapid eye movement sleep; SDB, Sleep-disordered breathing.

### Classification performances in analysis set

The neural network-based method yielded overall absolute accuracies of 84.6% (κ = 0.78), 82.3% (κ = 0.76), and 84.1% (κ = 0.78) in the training, validation, and test sets, respectively, during the 10-fold cross-validation. There was no meaningful difference in the test set (*n* = 105) performance between individuals recruited with SDB symptoms (*n* = 53) and age and sex-matched controls (*n* = 52) [83.9% (κ = 0.78) vs. 84.2% (κ = 0.78)]. In the analysis set, 24 children fulfilled the diagnostic criteria for pediatric OSA (AHI ≥ 1) after PSG. Out of these children, 15 were from the originally recruited SDB-symptomatic subgroup and the remaining 9 were from the asymptomatic control subgroup. There was similarly no meaningful difference in the test set performance between children with PSG quantified AHI ≥ 1 (*n* = 24) and those with AHI < 1 (*n* = 81) [82.9% (κ = 0.77) vs. 84.3% (κ = 0.78)].

**Figure 3:**
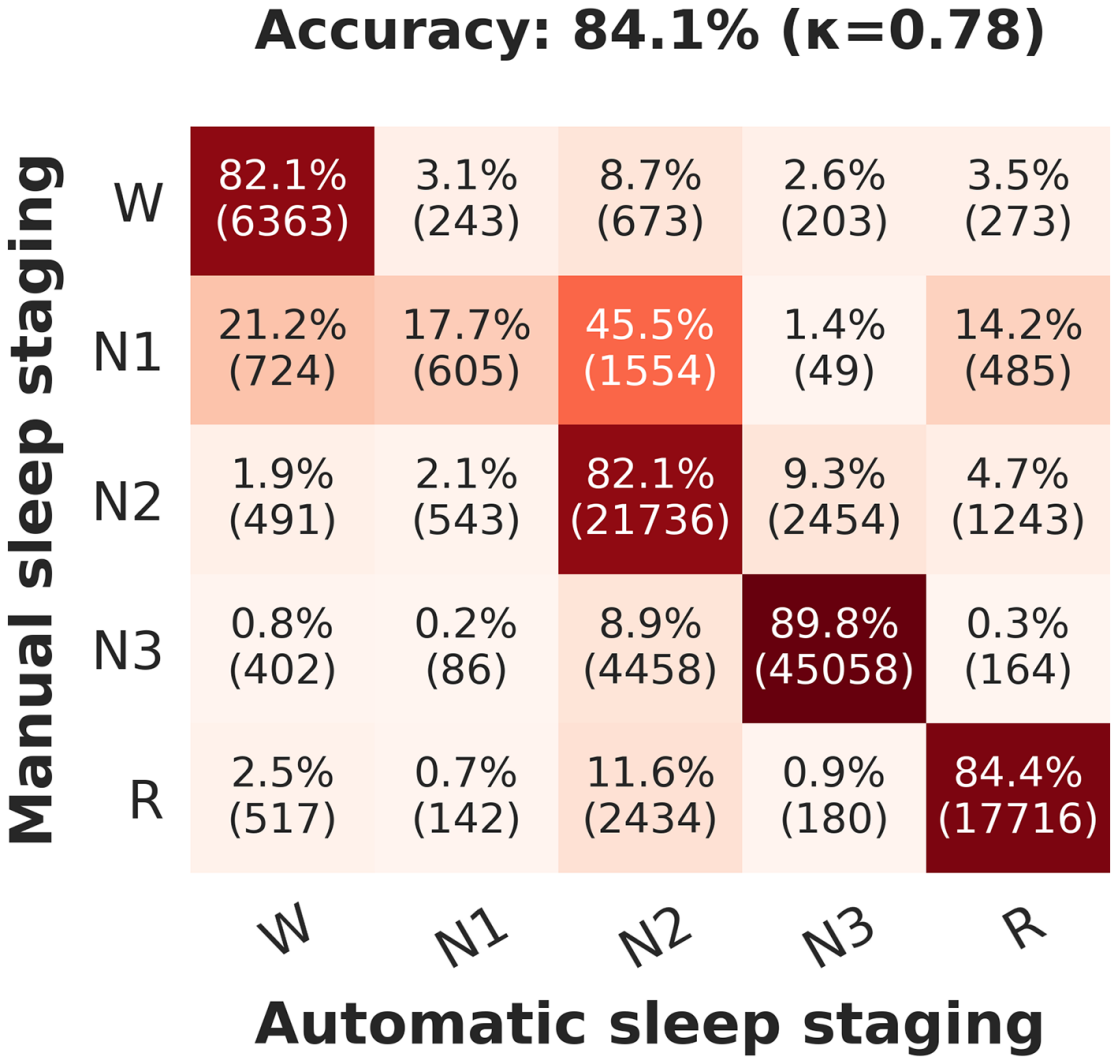
Confusion matrix of the test set classification performance aggregated across all 10 folds of the cross-validation in the primary analysis set (*n* = 105, total number of epochs = 108,796). Each row of the matrix represents the instances in the manual reference scoring while each column represents the instances in the neural network-predicted sleep scoring. The diagonal of the matrix shows all correct predictions. Values presented inside parentheses denote the number of epochs in each predicted class. W, Wakefulness; R, Rapid eye movement sleep; N1, N2, N3, Three different levels of non-rapid eye movement sleep; κ, Cohen’s kappa coefficient.

**Figure 4:**
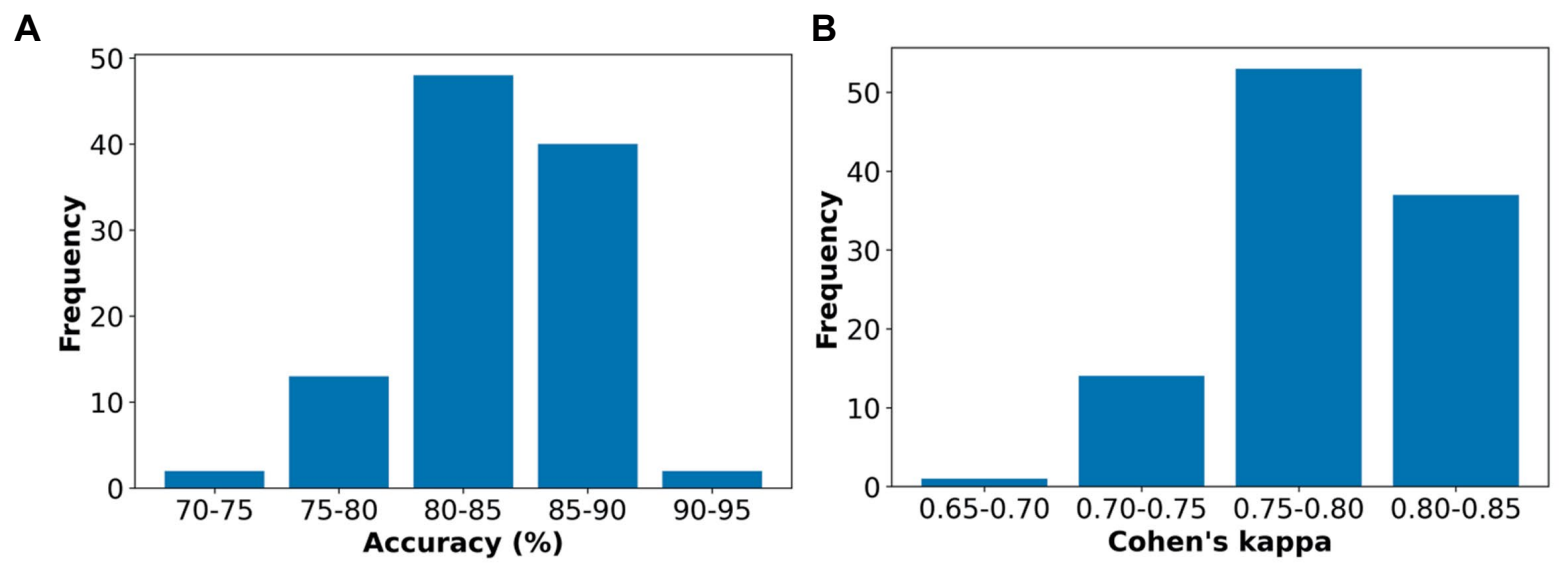
A summary of the individual-level automatic sleep stage classification performances: **(A)** Accuracies in percentage (range: 73.3%–90.4%) and **(B)** Cohen’s kappa (Range: 0.69 to 0.84) of all children comprising the aggregated test set (*n* = 105) across the 10 folds during cross-validation.

Considering the class-specific performance of the deep learning-based method, stage N1 had the lowest prediction accuracy of 17.7%, while the N3 stage attained the highest accuracy of 89.8% in the test set. Figure 3 presents the confusion matrix of the test set classification performance aggregated across all 10 folds of the cross-validation in the analysis set (*n* = 105, total number of epochs = 108,796). Figure 4 shows a summary of the individual-level automatic sleep stage classification performances of all children comprising the aggregated test set across the 10 folds during cross-validation (i.e., analysis set, *n* = 105). In the aggregated test set, our algorithm distinguished sleep epochs from wake epochs (references are based on manual scoring) with a sensitivity of 97.9% and a specificity of 82.1%. Table 3 presents detailed stage-wise classification performance metrics (i.e., sensitivity, specificity, positive predictive value, and negative predictive value) in the aggregated test set (*n* = 105).

**Table 3:** Detailed stage-wise classification performance metrics in the test set aggregated across the 10-fold cross-validation in the primary analysis set (*n* = 105).

Sleep stage	Sensitivity	Specificity	PPV	NPV
W	82.1%	97.9%	74.9%	98.6%
N1	17.7%	99.0%	37.4%	97.4%
N2	82.1%	88.9%	70.4%	93.9%
N3	89.8%	95.1%	94.0%	91.6%
R	84.4%	97.5%	89.1%	96.3%

W, Wakefulness; R, Rapid eye movement sleep; N1, N2, N3, Three different levels of non-rapid eye movement sleep; PPV, Positive predictive value; NPV, Negative predictive value.

The test set accuracies obtained for four- and three-stage classifications were 85.4% (κ = 0.80) [W: 81.8%, N1 + N2: 85.2%, N3: 85.5%, R: 86.9%] and 92.6% (κ = 0.84) [W: 79.8%, N1 + N2 + N3: 95.3%, R: 87.3%] respectively.

### Performance relative to intra- and inter-rater agreement in comparison set

The classification model retrained using the whole analysis set and evaluated on the comparison set for the purpose of comparing the automatic scoring with different manual scorings yielded an overall training accuracy of 87.2% (κ = 0.81) and an overall accuracy of 84.5% (κ = 0.78) in the unseen test set (i.e., the comparison set).

**Figure 5:**
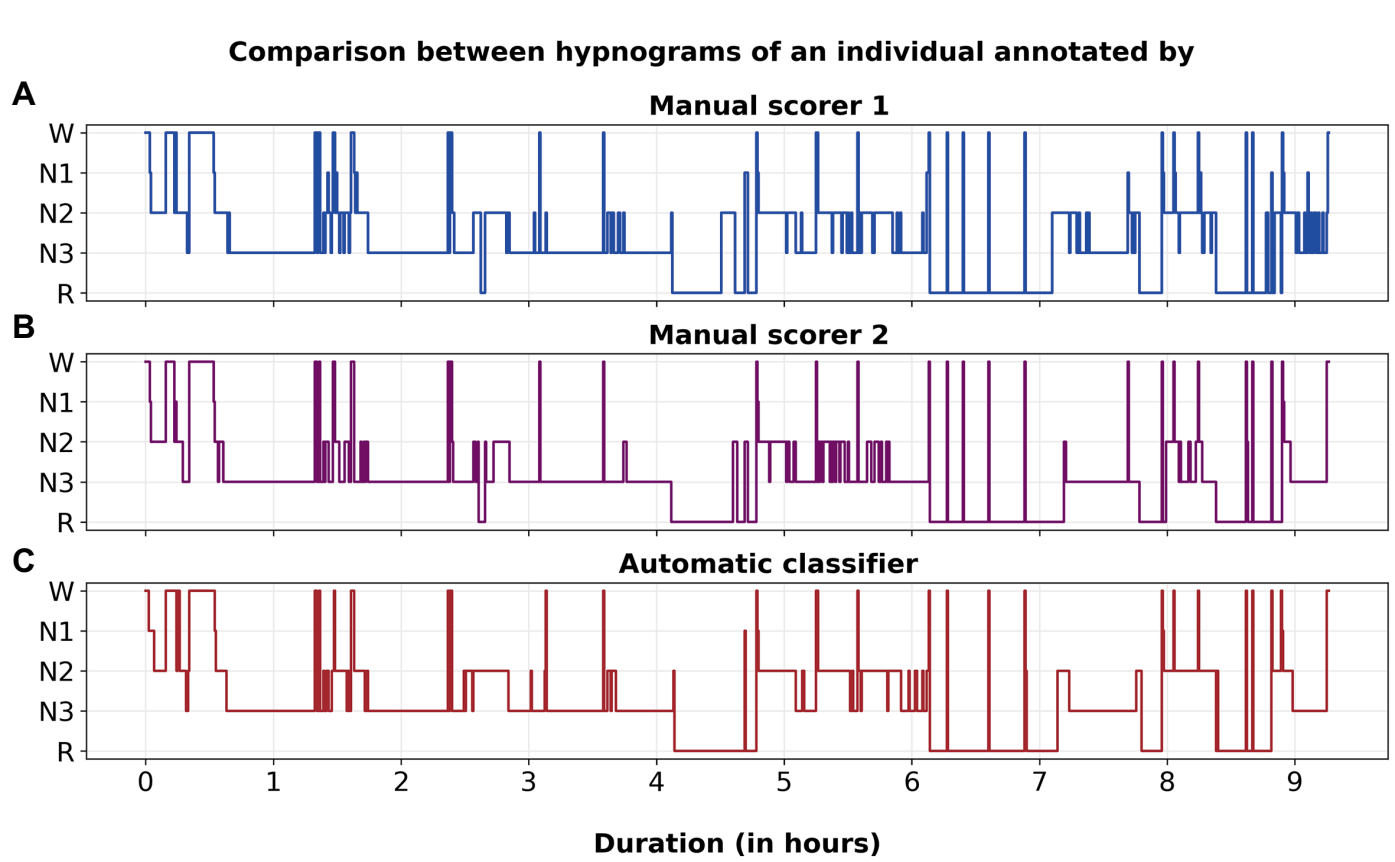
An example comparison between hypnograms of an individual annotated by **(A)** Manual scorer 1, **(B)** Manual scorer 2, and **(C)** Automatic classifier. The performance of the automated classifier in this individual was close to the population average (i.e., κ = 0.78 with manual scorer 1 and κ = 0.77 with scorer 2).

The overall inter-rater reliability between the two manual scorers was 84.6% (κ = 0.78) in the comparison set and the neural network-based automatic approach achieved similar agreements with scorers individually: 83.4% (κ = 0.76) and 82.7% (κ = 0.75). The intra-rater scoring consensuses were highest for sleep stage R for both scorers. In contrast, inter-rater agreements were highest for N3. As expected, the intra- and inter-rater agreements were lowest for N1 (Table 4). The neural network approach agreed with at least one of the manual scorers in 89.8% of the epochs. Similarly, when considering only the epochs with a scoring consensus between the manual scorers, 90.4% (κ = 0.86) of those epochs were also scored as the same sleep stage by the automatic classifier. The sleep stage-specific agreement in this instance were W: 88.2%, N1: 28.4%, N2: 93.0%, N3: 91.1%, and R: 89.7%. Figure 5 illustrates an example comparison between hypnograms of an individual annotated by manual scorers and the automatic classifier. The performance of the automated classifier in this individual was close to the population average (i.e., κ = 0.78 with manual scorer 1 and κ = 0.77 with scorer 2).

**Table 4:** Intra-rater and inter-rater reliability metrics for individual and overall sleep stage comparisons between manual scorers and the automatic method in a holdout subset of *n* = 10 (i.e., the comparison set).

	Intra-rater agreement: S1	Intra-rater agreement: S2	Inter-rater agreement: S1 versus S2	Inter-rater agreement: S1 versus Auto	Inter-rater agreement: S2 versus Auto
W	88.6%	89.6%	83.6%	80.8%	81.3%
N1	44.3%	63.1%	32.6%	24.9%	14.2%
N2	77.3%	81.4%	72.7%	83.8%	87.3%
N3	91.5%	92.5%	91.5%	87.7%	89.5%
R	92.7%	93.0%	90.7%	86.7%	86.2%
Overall	87.5%	89.3%	84.6%	83.4%	82.7%
κ	0.82	0.85	0.78	0.76	0.75
Remark	Almost Perfect	Almost Perfect	Substantial	Substantial	Substantial

Agreements between manual and automatic classifications were calculated using manual scoring as the reference. Agreements between manual classifications were obtained by averaging agreements calculated with each manual classification as the reference. The remarks on the agreements are based on the guidelines by Landis and Koch (61) for Cohen’s kappa values. S1, Human scorer 1; S2, Human scorer 2; Auto, Automatic method; κ, Cohen’s kappa coefficient; W, Wakefulness; R, Rapid eye movement sleep; N1, N2, N3, Three different levels of non-rapid eye movement sleep.

## Discussion

The overarching aim of this study was to develop a deep learning-based automatic sleep stage classification system for preadolescent children. As such, we developed a combined CNN-LSTM architecture utilizing a dataset containing overnight PSGs of Icelandic preadolescent children with SDB symptoms and age and sex-matched controls. The cross-validated sleep stage classification performance was evaluated with a 3-channel input (i.e., frontal EEG + EOG + chin EMG). In addition, to further evaluate the performance relative to human scoring and to examine the reliability of the model, we conducted a separate intra- and inter-rater agreement analysis in a subset (*n* = 10) of data with repeated scorings from two expert human scorers. Overall, our algorithm achieved a high classification accuracy and substantial agreement with both manual scorers. The performance metrics compared well with previous automated sleep staging methods and with inter-rater reliability between manual scorers both in this population and those reported in the literature (14). Moreover, the individual-level automatic classification accuracies and kappa values were consistent across both children with SDB symptoms and non-diseased controls. These findings indicate that our model enables accurate and reliable automatic sleep stage classification for preadolescent children.

In the present study, the classification performance metrics of all sleep stages except for N1 were excellent (Figure 3; Table 3), and the overall performance of this method compares well to the previously published studies involving pediatric populations (24–29, 40, 42, 44). However, direct comparison to previous studies is difficult due to different datasets and age variations. Previously, Huang et al. (25) adopted a timestamp-based segmentation strategy with a deconvolutional neural network for automatic sleep staging in children aged 5–10 years and achieved an accuracy of 84.3%. However, a considerably smaller dataset (*n* = 21) and a complex 11-channel input (i.e., 6 EEG + 2 EOG + 3 EMG) were used in that study (Table 5). In comparison, our study achieved a similar performance with a larger pediatric cohort using only a 3-channel input. In parallel with the development of our work, other studies have also focused on sleep staging including pediatric patients and have demonstrated similar performance metrics (40, 42, 44). Notably, Wang et al. (42) achieved high classification performance (with slightly lower kappa values compared to the present study when using a similar 3-channel input) with a modularized network utilizing a clinical pediatric dataset of 344 SDB patients with age 2–18 years (Table 5). Similarly, Phan et al. (44) demonstrated that different deep learning-based algorithms with good performance in adults also generalized well to 5–10-year-old children with SDB in the Childhood Adenotonsillectomy Trial dataset (62) (Table 5). Likewise, a large-scale study conducted by Perslev et al. (40) utilized multiple adult and pediatric datasets (i.e., PSG recordings from 15,660 participants of 16 clinical studies, including PSGs from 2 public pediatric sets) to train and evaluate a U-net architecture and attained high sleep stage classification accuracies. While our algorithm achieves similar or slightly higher performance to these previous studies, it makes two important unique contributions. Firstly, our study is the first to evaluate and demonstrate equivalent performance in children with both suspected SDB and community controls, thus demonstrating this important aspect of generalizability. Secondly, we specifically focus on preadolescent children, which are either not represented or are under-represented in previous works. This not only confirms the generalizability of such approaches to this age group; but also provides a tool to investigate sleep in this cohort in more detail. This is a period of substantial emotional and hormonal changes (52), and a better understanding of how sleep changes during this period would be highly desirable.

**Table 5:** Performance comparison of the present study with previous deep-learning-based pediatric sleep staging.

	Cohort size (*n*)	Community control group included (Y/N)	Age range	Stages	Accuracy [kappa (κ)]
Present work	115	Y	10 to 13 years	5 (W/N1/N2/N3/R)	84.1% (0.78)
				4 (W/N1 + N2/N3/R)	85.4% (0.80)
				3 (W/N1 + N2 + N3/R)	92.6% (0.84)
Jeon et al. (24)	218	N	-	3 (W/N1/N2)	92.2% (0.88)
Huang et al. (25)	21	N	5 to 10 years	5 (W/N1/N2/N3/R)	84.3% (−)
Wang et al. (42)	344	N	2 to 18 years	5 (W/N1/N2/N3/R)	82.6% (0.76)
				4 (W/N1 + N2/N3/R)	85.8% (0.79)
				3 (W/N1 + N2 + N3/R)	91.4% (0.81)
Phan et al. (44)	1,216	N	5 to 10 years	5 (W/N1/N2/N3/R)	88.8% (0.85)

Only studies utilizing deep learning techniques with EEG-based channels and specifically targeted at pediatric populations excluding infants are included. Y, Yes; N, No.

Our algorithm also performed comparably with the state-of-the-art sleep staging methods developed for adults (22, 23, 30–39, 41, 43, 45), which typically achieve kappa performance in the range of 0.67–0.87 (48–51). We previously demonstrated that a similar CNN-LSTM architecture for sleep staging works well in adult populations and outperformed previously published methods at the time (22). The accuracy and kappa values achieved in the present study considering a preadolescent cohort are almost identical to the performance metrics obtained in adult cohorts utilized by Korkalainen et al. (22). Therefore, our findings confirm that the considered architecture generalizes well to preadolescent children with SDB and non-diseased controls.

The inter-rater agreements achieved in this dataset are comparable to the consensus between manual scorers, where kappa is typically 0.76–0.78 in adult populations (14, 17). Considering the separate inter-rater reliability analysis conducted in the comparison set, the sleep stage-specific agreements obtained for the automatic method well exceeded the consensus between the two manual scorers in scoring sleep stage N2; were near-identical for W, N3, and R; but were modestly lower for stage N1 [possibly reflecting the relatively small (3.1%) proportion of N1 in this dataset] (Table 4). Nonetheless, our neural network-based approach showed substantial agreements (61) with both manual scorers and matched the concordance between human scorers. Moreover, the automatic approach matched with at least one of the two manual scorers in 89.8% of the epochs, while the match percentage between the manual scorers was only 84.6%, further emphasizing the reliability of the proposed algorithm relative to manual scoring.

Incorporating a reliable and accurate deep learning-based automatic sleep staging system to support the current clinical procedure could significantly benefit pediatric sleep disorder diagnosis. As elucidated in several studies (11–16), the traditional sleep scoring may lack adequate inter-rater reliability and manifest high variability. However, once trained, deep learning-based approaches, including the proposed model, would always classify sleep stages uniformly for the same data. This can be a substantial advantage of our model compared to visual sleep scoring as it eliminates limitations such as human-scorer vigilance-related errors. Finally, manual scoring is laborious, time-consuming, and expensive. The proposed method can perform quickly once trained (i.e., typically well within a minute per overnight study) and would significantly improve the efficiency of the sleep stage classification process.

The main performance limitation of the proposed algorithm is the low classification performance and inter-rater agreements of stage N1 (Figure 3; Tables 3, 4). As expected, the overall accuracy in classifying stage N1 was poor (only 17.7%), and N1 sleep was most frequently confused with N2, and then with wake (Figure 3). One explanation for this is the relatively small amount of N1 epochs in the dataset (only 3.1%) and therefore the algorithm is relatively poorly trained on this stage. However, inter-human-rater agreements for N1 were similarly low in both our study and published literature where N1 agreements range between κ = 0.19–0.31 (11, 12). This suggests that even for experienced manual sleep scorers, N1 is the hardest sleep stage to identify.

The mean (± SD) total sleep time (TST) of 479.6 ± 54.1 min observed in this dataset is lower than the typical average TST in this age group (63, 64). Similarly, we identified that the proportion of R sleep is slightly lower than what is usually observed in preadolescent children (64). There are two possible explanations for this discrepancy. Firstly, for other scientific purposes, the children wore a double EEG setup with two devices, a scoop cannula over their mouth, and an additional electrodermal activity (EDA) sensor (65); and this may have caused them to wake up earlier than usual and take the equipment off and consequently may have affected the TST and R sleep proportion. Second, this study was performed in Iceland during the summer months with an unusual amount of daylight, which may also have possibly caused early awakenings.

The study population consisted of Icelandic preadolescent children with symptoms of SDB (*n* = 59) and age and sex-matched controls (*n* = 56). However, there were no meaningful differences in the demographic characteristics between these two subgroups. Post PSG, 26 children fulfilled the diagnostic criteria for pediatric OSA (AHI ≥ 1) [17 from the SDB symptomatic subgroup and 9 from the asymptomatic control subgroup]; out of which, only one individual was deemed to have moderate pediatric OSA (AHI ≥ 5). Severe OSA was not found, and the study population did not explicitly include children with other sleep disorders. Different sleep disorders have distinct characteristics and could cause significant sleep architectural changes and deteriorated sleep quality. For example, OSA patients usually have more light sleep stages and less N3 and R sleep (66), whereas narcolepsy patients usually have fragmented sleep and abnormal and frequent sleep stage R occurrences (67). As such, further investigations are required to confirm the generalizability of our algorithm in these other groups, including those with more moderate and severe OSA. We believe these results must be generalized with caution to other heterogeneous clinical populations or centers internationally where participant characteristics may vary substantially; also to children with age range out of that in the present study. Similarly, this was well-curated scientific data. However, in practice, the algorithm would need to cope with artefact typical of clinical sleep studies; and further validation is required to examine the performance of this algorithm in these conditions. Further, it is likely that modern deep learning-based automated sleep classifiers have already achieved near-saturated performance metrics (68). Therefore, to be incorporated into clinical practice, future studies must focus more on improving the generalizability, reliability, uncertainty quantification, and interpretability of deep learning-based sleep staging models (44, 48, 51). Finally, to date, this and other studies focused on the classification of sleep stages without consideration of arousal events. Given the significant physiological overlap between arousal and wake stage, there are likely to be significant advantages to incorporating arousal event scoring within the same algorithm as sleep stage classification.

## Conclusion

Pediatric sleep disorders are prevalent, and manual sleep stage classification has significant challenges. As such, incorporating an accurate and reliable automatic sleep staging method in clinical practice would greatly assist in improving the efficiency of pediatric sleep disorder diagnosis. The proposed deep learning-based classification algorithm enables fast, accurate, and reliable automatic sleep staging based on frontal EEG, EOG, and chin EMG signals in preadolescent children. Our findings favor the utility of deep learning-based approaches for sleep staging over the traditional manual method.

## Data availability statement

The data analyzed in this study is subject to the following licenses/restrictions: This dataset is subject to strict ethical restrictions and cannot be shared or distributed without prior approval from the corresponding ethical committee. Access to the data is limited to individuals who have been granted explicit permission for its use. Requests to access these datasets should be directed to PS, p.somaskandhan@uq.edu.au.

## Ethics statement

The studies involving human participants were reviewed and approved by Ethical Committee of Landspitali—the National University Hospital of Iceland and the National Bioethics Committee of Iceland (VSN 18–206). Written informed consent to participate in this study was provided by the participants’ legal guardian/next of kin.

## Author contributions

JT, TL, PT, and HK devised the project and the main conceptual ideas for the analyses. SÞS and MC designed and carried out the initial study and provided the data for this analysis. SS, EA, KÓ, and MS contributed to data interpretation. PS, HK, PT, TL, and JT carried out the data preparation and the analyses. PS drafted the manuscript and prepared the figures and tables. All authors contributed to the article and approved the submitted version.

## Funding

This study was funded by Nordforsk (NordSleep, no. 90458) *via* Business Finland (no. 5133/31/2018) and *via* the Icelandic Centre for Research, the Icelandic Research Fund (no. 174067), the Landspitali University Hospital Science Fund 2019 (no. 893831), the European Union’s Horizon 2020 Research and Innovation Programme (grant no. 965417), the National Health and Medical Research Council (NHMRC) of Australia (project nos. 2001729 and 2007001), the Academy of Finland (project no. 323536), the Research Committee of the Kuopio University Hospital Catchment Area for the State Research Funding (project nos. 5041794 and 5041803), and the Finnish Anti-Tuberculosis Association and the Research Foundation of the Pulmonary Diseases. The birth cohort study was funded by the European Commission: (a) under the 6th Framework Program (FOOD-CT-2005-514000) within the collaborative research initiative “EuroPrevall” and (b) under the 7th Framework Program (FP7-KBBE-2012-6; grant agreement no. 312147) within the collaborative project “iFAAM.” Additional funds were received by the Icelandic birth cohort center from Landspitali University Hospital Science Fund, and GlaxoSmithKline Iceland. The funders were not involved in the study design, collection, analysis, interpretation of data, the writing of this article or the decision to submit it for publication.

## Conflict of interest

The authors declare that the research was conducted in the absence of any commercial or financial relationships that could be construed as a potential conflict of interest.

## Publisher’s note

All claims expressed in this article are solely those of the authors and do not necessarily represent those of their affiliated organizations, or those of the publisher, the editors and the reviewers. Any product that may be evaluated in this article, or claim that may be made by its manufacturer, is not guaranteed or endorsed by the publisher.
